# normGAM: an R package to remove systematic biases in genome architecture mapping data

**DOI:** 10.1186/s12864-019-6331-8

**Published:** 2019-12-30

**Authors:** Tong Liu, Zheng Wang

**Affiliations:** Department of Computer Science, University of Miami, 1365 Memorial Drive, P.O. Box 248154, Coral Gables, FL 33124 USA

**Keywords:** Genome architecture mapping, GAM, Hi-C, 3D genome structure, Normalization, Systematic biases

## Abstract

**Background:**

The genome architecture mapping (GAM) technique can capture genome-wide chromatin interactions. However, besides the known systematic biases in the raw GAM data, we have found a new type of systematic bias. It is necessary to develop and evaluate effective normalization methods to remove all systematic biases in the raw GAM data.

**Results:**

We have detected a new type of systematic bias, the fragment length bias, in the genome architecture mapping (GAM) data, which is significantly different from the bias of window detection frequency previously mentioned in the paper introducing the GAM method but is similar to the bias of distances between restriction sites existing in raw Hi-C data. We have found that the normalization method (a normalized variant of the linkage disequilibrium) used in the GAM paper is not able to effectively eliminate the new fragment length bias at 1 Mb resolution (slightly better at 30 kb resolution). We have developed an R package named normGAM for eliminating the new fragment length bias together with the other three biases existing in raw GAM data, which are the biases related to window detection frequency, mappability, and GC content. Five normalization methods have been implemented and included in the R package including Knight-Ruiz 2-norm (KR2, newly designed by us), normalized linkage disequilibrium (NLD), vanilla coverage (VC), sequential component normalization (SCN), and iterative correction and eigenvector decomposition (ICE).

**Conclusions:**

Based on our evaluations, the five normalization methods can eliminate the four biases existing in raw GAM data, with VC and KR2 performing better than the others. We have observed that the KR2-normalized GAM data have a higher correlation with the KR-normalized Hi-C data on the same cell samples indicating that the KR-related methods are better than the others for keeping the consistency between the GAM and Hi-C experiments. Compared with the raw GAM data, the normalized GAM data are more consistent with the normalized distances from the fluorescence in situ hybridization (FISH) experiments. The source code of normGAM can be freely downloaded from http://dna.cs.miami.edu/normGAM/.

## Introduction

The Hi-C technique uses different restriction enzymes and proximity-based ligation to capture genome-wide chromatin interactions [1, 2], which provides large-scale maps of the three-dimensional (3D) architecture of the whole genome. Similar to the Hi-C experiments [1], the genome architecture mapping (GAM) experiments [3] can also capture genome-wide chromatin proximities. However, GAM has the following advantages compared with Hi-C [1]: (1) GAM only needs ultrathin cryosectioning instead of ligation; (2) GAM experiments can detect triplet contacts between multiple chromatin regions more effectively than the Hi-C experiments; (3) GAM only needs hundreds of cells compared with millions of cells needed in population Hi-C experiments [2–4]. Although it is not the focus of this study, it is important to mention that single-cell Hi-C technique has been invented [5, 6] to capture the DNA proximities of individual cells, based on which 3D genome structures of individule cells can be reconstructed [7]. GAM has been included in the 4D Nucleome (4DN) Network [8], can be visualized using 3D Genome Browser [9], and has been used to assess the accuracy of 3D chromatin reconstructions [10]. All of the interacting capture techniques including Hi-C, the variants of Hi-C such as HiChIP [11] and SPRITE [12], and GAM can generate whole-genome contact maps, which can be used to reconstruct chromatin 3D architectures [13–16].

In the GAM experiment, a slice or an extra thin layer is cut at a random direction out of a single nucleus. By performing the same action on hundreds of nuclei at random orientations, GAM can gather hundreds of thin DNA layers. Each of these layers is referred as a nuclear profile (NP) that contains chromatin fragments that may be from different regions of a chromosome or even from different chromosomes [3]. After thin cryosections, five steps are performed in order to get the GAM DNA reads [3]: (1) fixing chromatins; (2) protein digestion and crosslink removal; (3) fragmentation (longer fragments are split into sub-fragments); (4) universal adaptor addition; and (5) amplification of the DNA reads. The DNA reads from the same NP have the same type of adaptor. Therefore, after being sequenced, all reads are mapped back to the reference genome to obtain their genomic locations.

**Fig. 1:**
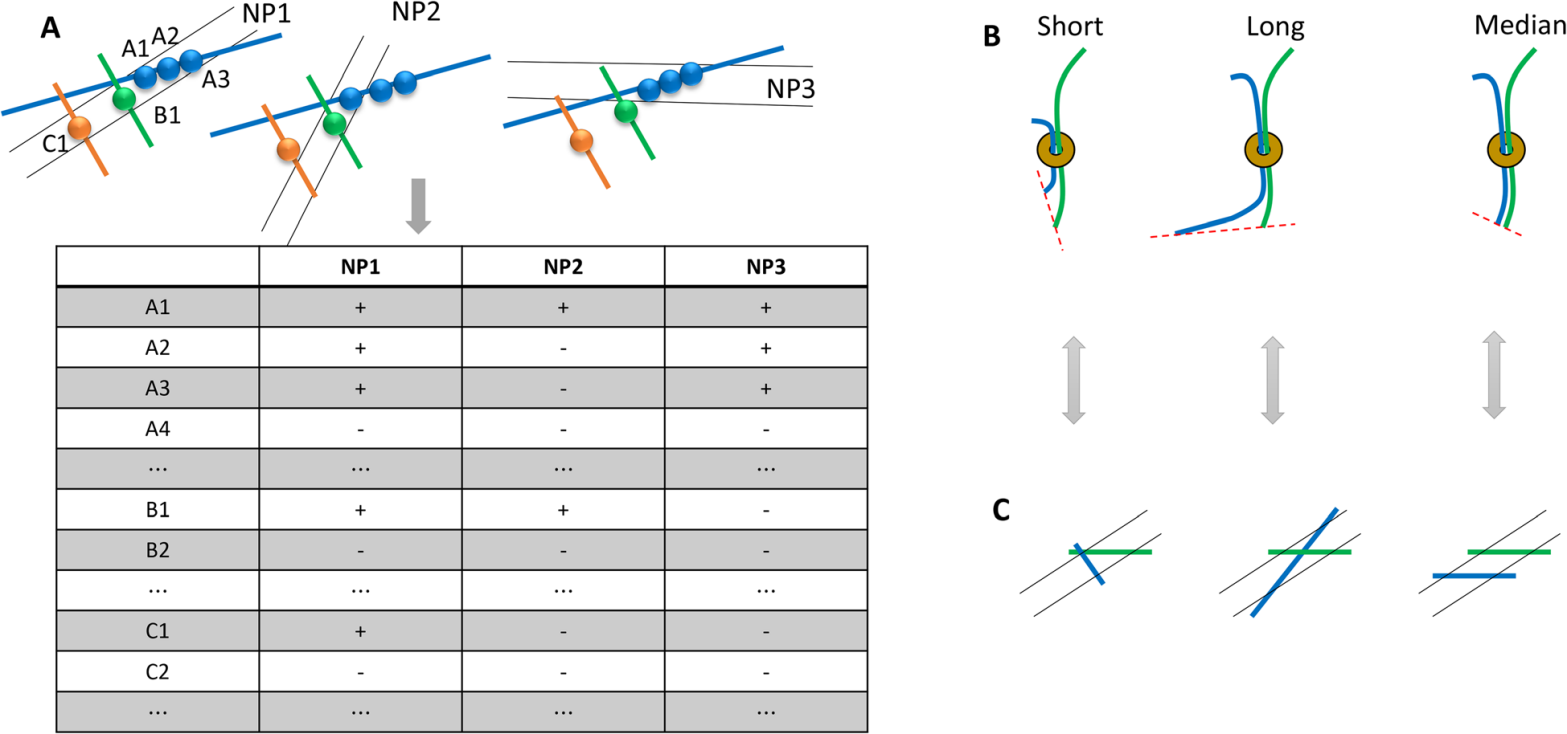
**a** An illustration of the calculation of fragment biases we newly found. The bins (i.e., A1, A2, A3, B1, and C1) have the same lengths that are equal to the resolution of interest. The plus sign in the table indicates that the bin is detected in the current NP. The negative sign indicates that the bin is not detected in the NP. **b** An illustration of distance biases between restriction sites in the Hi-C experiments. Different fragment lengths influence ligation efficiency in the Hi-C experiments. **c** The corresponding fragment length biases in the GAM experiments. Compared with the last NP, the lengths of blue and green fragments in the first two NPs are unevenly detected

After these steps, a co-segregation matrix is generated. Figure 1a, up shows three example NPs, each containing one or more bins (the orange, green, and blue colored beads in Fig. 1); and Fig. 1a, bottom shows the co-segregation matrix generated from these NPs. The number of columns in the co-segregation matrix is equal to the number of NPs, whereas the number of rows in the co-segregation matrix is equal to the number of bins of the whole genome. For example, the top three plus signs in the column of NP1 indicate that the bins A1, A2, and A3 are all detected in NP1. The top one plus sign in the column of NP2 indicates A1 is detected in NP2. However, A2 and A3 are not detected in NP2.

From the co-segregation matrix, the GAM contact matrix can be generated. The entries in the raw GAM contact matrices are obtained by using linkage disequilibrium *D*_*AB*_ = *f*_*AB*_ − *f*_*A*_*f*_*B*_ [3], where *f*_*AB*_ is equal to the frequency of nuclear profiles in which two bins *A* and *B* are simultaneously detected; *f*_*A*_ or *f*_*B*_ is the frequency of nuclear profiles in which the locus *A* or *B* is detected. Therefore, it is possible that *D*_*AB*_ is less than zero, which makes the raw GAM contact matrix negative.

There are multiple types of systematic biases found in the raw population Hi-C data [17, 18] including the biases caused by different distances between successive restriction sites, GC content, and mappability. We recently confirmed that these biases also existed in raw single-cell Hi-C data [19]. GAM raw data were also found to have the GC content and mappability biases as mentioned in the paper that introduced the GAM technique [3]. Moreover, that paper [3] has mentioned that raw GAM data also have the window (or bins) detection frequency bias [3] that will be defined later in Methods section.

Windows and bins are defined interchangeably in this study as chromatin segments with the same length; and the window/bin size is also referred as the resolution, which is used to evenly split an entire chromatin. In contrast, we define fragments as the chromatin segments that are cut out based on the restriction enzymes in Hi-C, for example, the two fragments (blue and green) in Fig. 1b. We also define fragments as the chromatin segments existing in each of the NPs in GAM, for example, the blue and green fragments in Fig. 1c. The relationship between a fragment and a window/bin is that a fragment is usually longer and may contain multiple bins.

However, the GAM paper [3] misses an important bias that is caused by different lengths of fragments, named by us hereafter as the fragment length bias. This bias is similar to the bias of distances between restriction sites in raw Hi-C data. These biases (window detection frequency and fragment length) need to be removed to ensure that the significant interactions found in contact matrices are not resulted from the systematic biases.

In this paper, we demonstrate the existence of the fragment length bias in raw GAM data caused by different fragment lengths from random slicing, which was not discussed in [3]. A software package named normGAM has been developed that contains five methods for normalizing raw GAM data including normalized linkage disequilibrium (NLD) [3], vanilla coverage (VC) [1], sequential component normalization (SCN) [20], iterative correction and eigenvector decomposition (ICE) [21], and Knight-Ruiz 2-norm (KR2) [2, 22].

## Materials and methods

### GAM and hi-C data

We downloaded the raw co-segregation GAM data for 408 nuclear profiles (NPs) from GEO (GSE64881) at two resolutions (i.e., 1 Mb and 30 kb). The raw intra-chromosomal (cis) GAM contact matrices were generated using the co-segregation data as input.

We downloaded the raw Hi-C reads for mouse embryonic stem (mES) cells from GEO (GSE35156) and combined two HindIII replicates to generate the raw Hi-C contact matrices at 1 Mb resolution.

### Definition of the systematics biases

#### GC content bias and mappability bias

The GC content and mappability biases in GAM are the same as in Hi-C [17, 18]. Therefore, detailed definitions will not be shown here. The GC content bias of a certain bin is the GC content of its DNA sequence. The mappability bias of a certain bin is generated in the same way as in scHiCNorm [19].

#### Window detection frequency bias

The existence of window detection frequency (WDF) bias in raw GAM data has been mentioned in [3] and is intuitively apparent because GAM cannot ensure that all of the bins are detected with the same frequency. Our analysis has proved the existence of this bias (data shown later), which indicates that the random orientations of the GAM cryosections cannot make all the bins to have the same chance to be detected in the NPs. For example, bin A1 is detected three times in Fig. 1a, A2 is detected two times, and A3 two times. Accordingly, we define their WDF biases as 3, 2, and 2.

#### Fragment length bias

The fragment length bias is based on similar idea as the window detection frequency bias. The window detection frequency bias is caused by the inconsistent frequencies for bins to be detected in the NPs, whereas the fragment length bias is also a type of bias for each bin but is caused by the inconsistent frequencies for fragments to be detected in the NPs. Our evaluation results will later prove that both two biases exist in raw GAM contact matrices.

For example, in Fig. 1a the fragment length for the blue chromatin segment is three bins based on NP1. For NP2, it is one bin, and three bins in terms of NP3. Therefore, we define the average fragment length of bin A1 as (3 + 1 + 3)/3 based on all of the three NPs. Similarly, we define the average fragment length of bin A2 as (3 + 0 + 3)/3 according to the three NPs. We use the average fragment lengths from all NPs as the fragment length bias of the bin. In our software, we omit the division of the number of NPs because this value is the same for all the bins. In this way, the fragment length biases of A1, A2, and A3 are 7, 6, and 6 in the example in Fig. 1.

The WDF bias and the newly found fragment length bias in the raw GAM data can be quantified by the *ld_nld_cis* function in our normGAM package. However, when a user is using the package to normalize raw GAM data, he/she does not need to quantify these biases in order to run the normalization algorithms. Instead, the algorithm can directly remove all the biases. The quantification of these biases is to prove the existence of the biases and to evaluate the performance of our bias-removing algorithms. If GC content and mappability biases at different resolutions are of interest, users can obtain the corresponding bias data for different reference genomes from our previous normalization method scHiCNorm [19].

### Normalization methods

The normalization methods developed for raw Hi-C contact matrices (e.g., KR [2, 22] and HiCNorm [18]) may not be directly used for raw GAM data because (1) the input of KR algorithm needs to be a non-negative symmetric matrix, but the raw GAM contact matrices may contain negative entries; (2) HiCNorm [18] and scHiCNorm [19] were designed for count data, but the raw GAM contacts were composed of real numbers. To address the first problem, we developed a new method KR2, the 2-norm of KR, which was able to handle negative contact matrices.

The Knight-Ruiz (KR) [22] algorithm was designed to balance a matrix. Given a non-negative symmetric matrix *D*, the algorithm tries to find a vector *x* such that

1 $$ \mathit{\operatorname{diag}}(x) Dx=e, $$

where *diag*(*x*) is a diagonal matrix converted from *x*, and *e* represents a vector of all ones.

Eq. (1) can be turned into:

2 $$ f\left({x}_{\ast}\right)=\mathit{\operatorname{diag}}\left({x}_{\ast}\right)D{x}_{\ast }-e=0. $$

We can obtain the iterative from eq. (2):

3 $$ {x}_{k+1}=\mathit{\operatorname{diag}}{\left(D{x}_k\right)}^{-1}e. $$

We can also use Newton’s method to get an alternative to eq. (3):

4 $$ {x}_{k+1}={x}_k-f\left({x}_k\right)/{f}^{\prime}\left({x}_k\right)={x}_k-{\left(\mathit{\operatorname{diag}}\left({x}_k\right)D+\mathit{\operatorname{diag}}\left(D{x}_k\right)\right)}^{-1}\left(\mathit{\operatorname{diag}}\left({x}_k\right)D{x}_k-e\right), $$

which can be rearranged as:

5 $$ \left(D+\mathit{\operatorname{diag}}{\left({x}_k\right)}^{-1}\mathit{\operatorname{diag}}\left(D{x}_k\right)\right){x}_{k+1}=D{x}_k+\mathit{\operatorname{diag}}{\left({x}_k\right)}^{-1}e. $$

Let *D*_*k*_ = *D* +  *diag* (*x*_*k*_)^−1^ *diag* (*Dx*_*k*_), which is symmetric as *D*. The next step is to use inner-outer iteration schemes with conjugate gradient method to solve eq. (5). When both sides of eq. (5) are premultiplied by *diag*(*x*_*k*_), we can get:

6 $$ \left({B}_k+\mathit{\operatorname{diag}}\left({B}_ke\right)\right){y}_{k+1}=\left({B}_k+I\right)e, $$

where *B*_*k*_ =  *diag* (*x*_*k*_)*Ddiag*(*x*_*k*_) and *y*_*k* + 1_ =  *diag* (*x*_*k*_)^−1^*x*_*k* + 1_. Eq. (6) can be solved using conjugate gradient iteration. More details about the initial values and stopping criteria can be found in [22]. The final balanced matrix can be obtained by *diag*(*x*)*Ddiag*(*x*). In summary, the original KR was designed for balancing a matrix in the 1-norm. Here, we present KR2 for balancing a GAM contact matrix in the 2-norm by taking the following three steps. We first conduct element-wise product by *D*_2_ = *D* · *D* and then run the original KR algorithm on *D*_2_ to get balanced matrix *D*_3_ = *KR*(*D*_2_). Finally, we obtain the normalized matrix *D*_*norm*_ = *D*_3_ ·  *sign* (*D*), where *sign*(*D*) denotes signed all-ones matrix; and the entries’ signs are consistent with the corresponding entries in *D*.

The vanilla coverage (VC) [1], sequential component (SCN) [20], and iterative correction and eigenvector decomposition (ICE) [21] normalization methods share similarities in their methodologies. The details of the three methods are given below. Each normalized entry is calculated by *D*_*ij*_/(*e*_*i*_*e*_*j*_), where *D*_*ij*_ is the entry in the raw contact matrix *D*.

As for VC, *e*_*i*_ is 1-norm of the *i*th row; and *e*_*j*_ is 1-norm of the *j*th column. As for SCN, *e*_*i*_ is 2-norm of the *i*th row; and *e*_*j*_ is 2-norm of the *j*th column. The original SCN also uses maximum iterations to reduce errors, but we have found that SCN performs better when conducting only one iteration on GAM data. Therefore, the normalization results from SCN in this work were all using one iteration.

As for ICE, *e*_*i*_ is 1-norm of the *i*th row divided by its mean value over non-zero bins; and *e*_*j*_ is 1-norm of the *j*th column divided by its mean value over non-zero bins. There are two stopping criteria in ICE including the maximum iteration and error tolerance. Based on our experience, it is difficult to achieve a pre-defined error tolerance (e.g., 1e-6) for all chromosomes; and when we set a large value (e.g., 6000) to the maximum iteration the normalized values for several chromosomes are extremely irregular. Therefore, we set it to 100 in this work. If the error is less than 1e-3, the algorithm will be terminated.

Normalized Linkage Disequilibrium (NLD) was implemented in the same way as described in [3]. The normalized *D*_*AB*_ is calculated by *D*_*AB*_/*D*_*max*_, and *D*_*max*_ is the theoretical maximum and defined as:

7 $$ {D}_{max}=\left\{\begin{array}{c}\mathit{\min}\left\langle {f}_A{f}_B,\left(1-{f}_A\right)\left(1-{f}_B\right)\right\rangle\ if\ {D}_{AB}<0\\ {}\ \mathit{\min}\left\langle {f}_B\left(1-{f}_A\right),{f}_A\left(1-{f}_B\right)\right\rangle\ if\ {D}_{AB}>0.\end{array}\right. $$

### Evaluation of the normalization methods

For each of the four biases (WDF, fragment length, mappability, and GC content), we sorted the chromatin bins in the whole genome (chr1 to chr19) based on their biases, stratified the whole bins of a genome into 20 sets, and then calculated the average number of GAM contacts between each pair of the 20 sets, resulting in a 20-by-20 heat map. We did the same operations on both the raw and normalized GAM contact matrices. A good normalization method can make the 20-by-20 heat map as smooth as possible, that is, the normalized 20-by-20 heat map is mostly occupied by one color.

Another evaluation measure is the Pearson’s correlation between the GAM data (both before and after normalization) and the four known biases. A good normalization method can achieve a very low correlation (i.e., close to zero) in terms of each of the four biases.

## Results and discussion

**Fig. 2:**
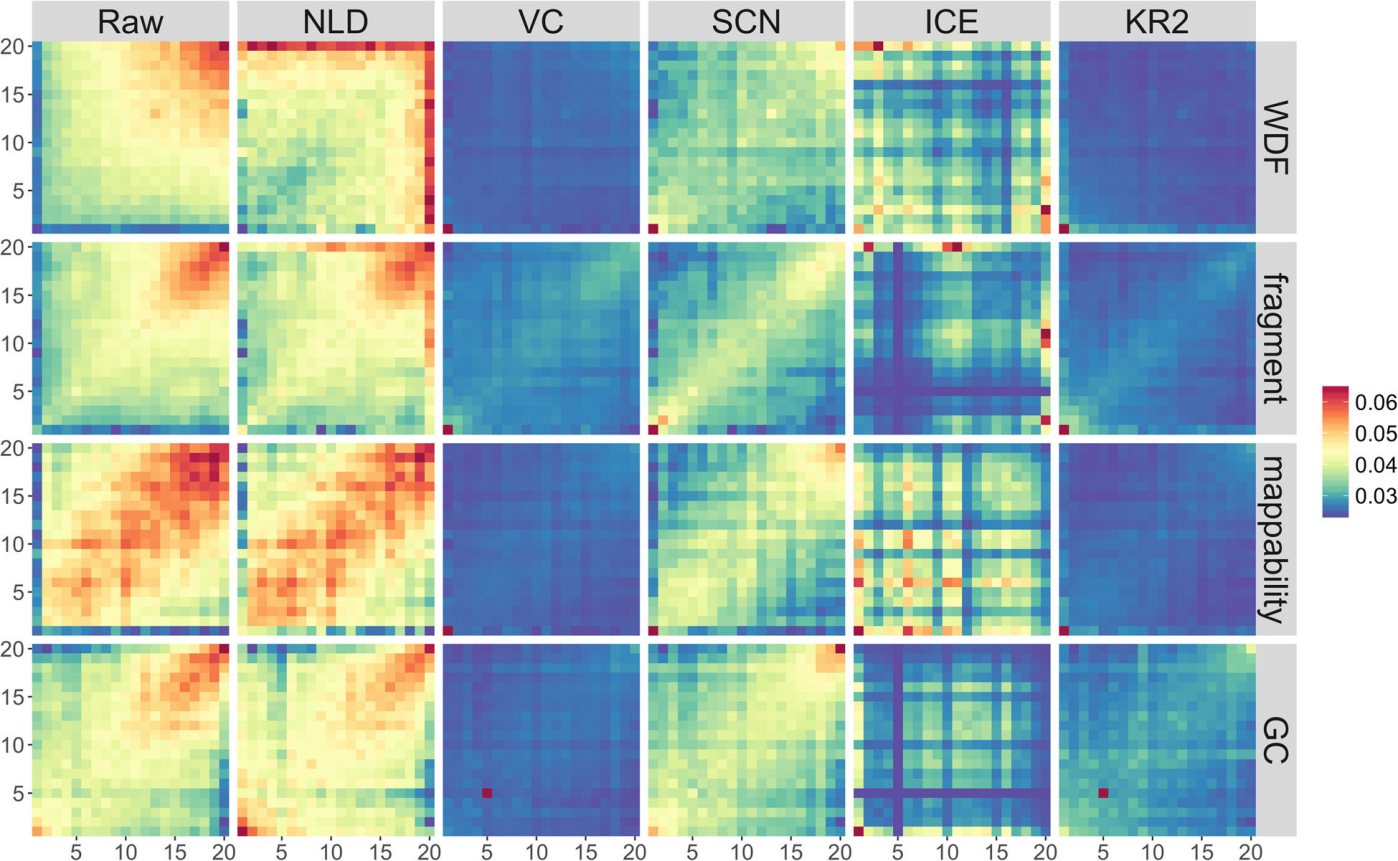
The 408 nuclear profiles at 1 Mb resolution showing the four systematic biases (i.e., WDF, fragment length, mappability, and GC content). Five normalization methods can eliminate the four biases at different efficiency levels

**Fig. 3:**
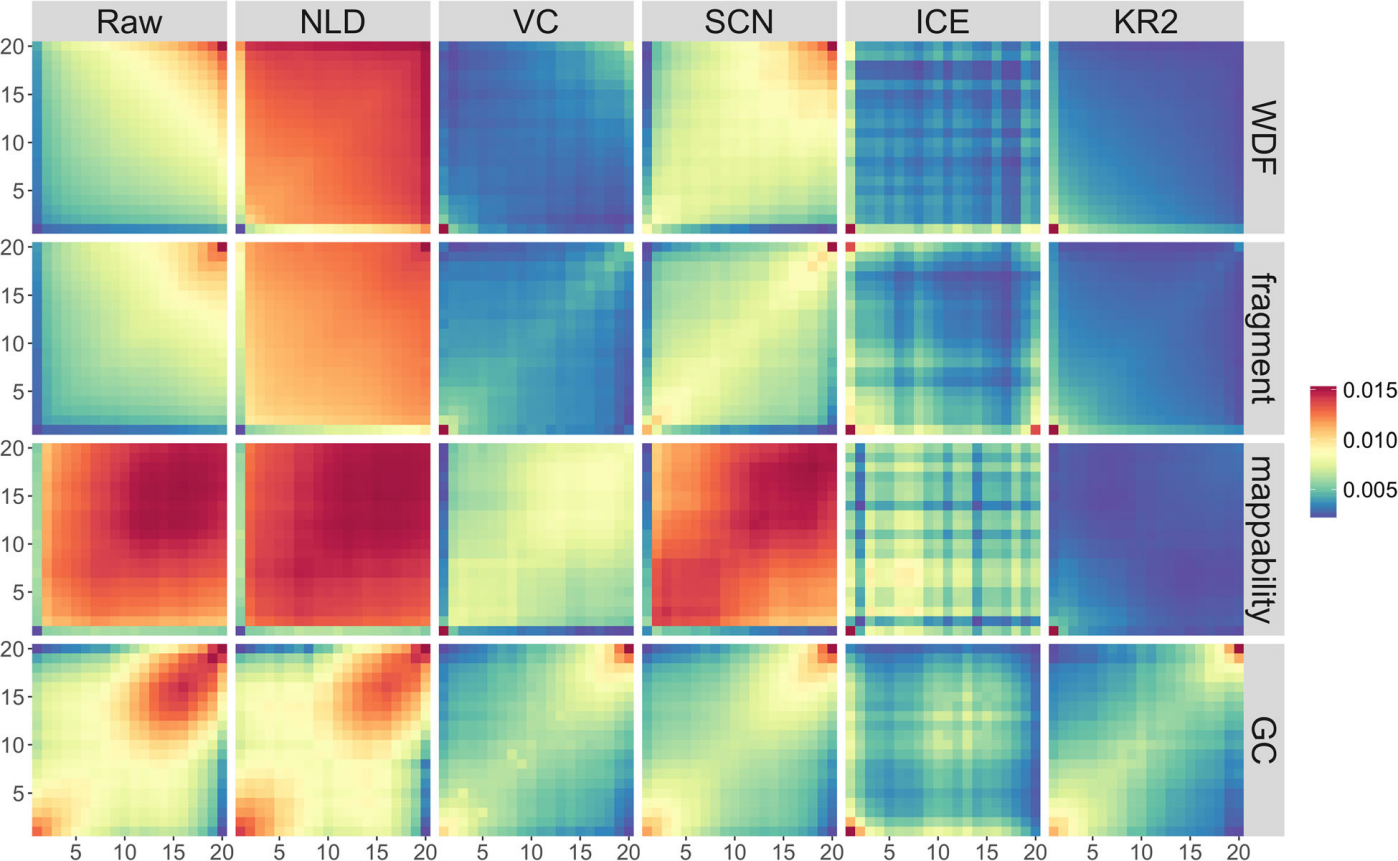
The 408 nuclear profiles at 30 kb resolution showing the four systematic biases (i.e., WDF, fragment length, mappability, and GC content). Five normalization methods can eliminate the four biases at different efficiency levels

We found that the new fragment length bias did exist in the raw GAM data at 1 Mb and 30 kb resolutions based on 408 NPs. Figure 2 is for 1 Mb resolution and Fig. 3 for 30 kb resolution. Compared with the blue regions with lower values, the red regions along the diagonal indicate the existence of biases. We did both Students’ t-tests and Wilcoxon singed-rank tests on the two data sets of WDF and fragment length biases at 1 Mb resolution for each of 19 chromosomes. The *p*-values from the two tests for all chromosomes are consistently lower than 1e-10, indicating that the two biases (WDF and fragment length) are significantly different from each other. Together with WDF, mappability, and GC content, we have detected four biases in the raw GAM data.

**Fig. 4:**
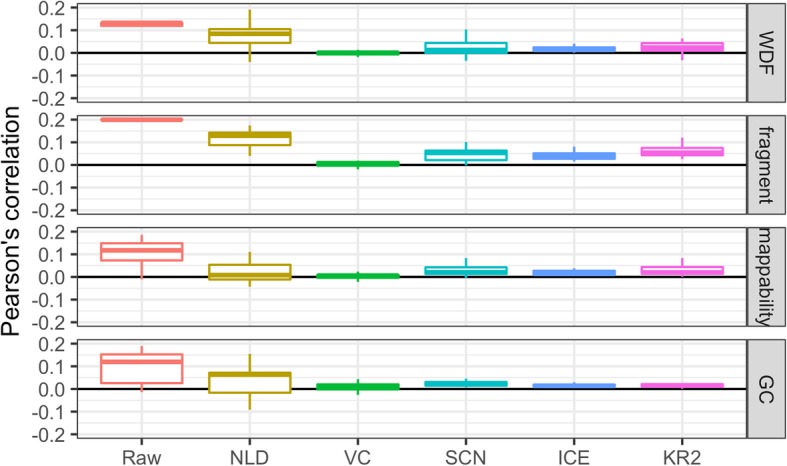
The Pearson’s correlation between the four systematic biases and the raw/normalized GAM data generated from 408 nuclear profiles at 1 Mb resolution

We evaluated the performance of the five normalization methods at 1 Mb and 30 kb resolutions. The normalization results are shown in Figs. 2, 3, and 4. Compared with the method we newly designed (KR2), the normalization method NLD that was previously used in the GAM paper [3] was not able to effectively eliminate fragment length, WDF, mappability, or GC content biases at 1 Mb resolution although its performance was better at 30 kb resolution for the biases of WDF and fragment length. All the five methods eliminated the four known biases in varying degrees, with two methods (i.e., VC and KR2) performing much better than the others at both resolutions.

We calculated the Pearson’s correlation between GAM data (both raw and normalized) and each of the four biases (Fig. 4), which showed that VC, ICE, and KR2 performed better than the other two methods. Notice that a zero or close-to-zero correlation indicates complete or close-to-complete removal of the bias.

**Fig. 5:**
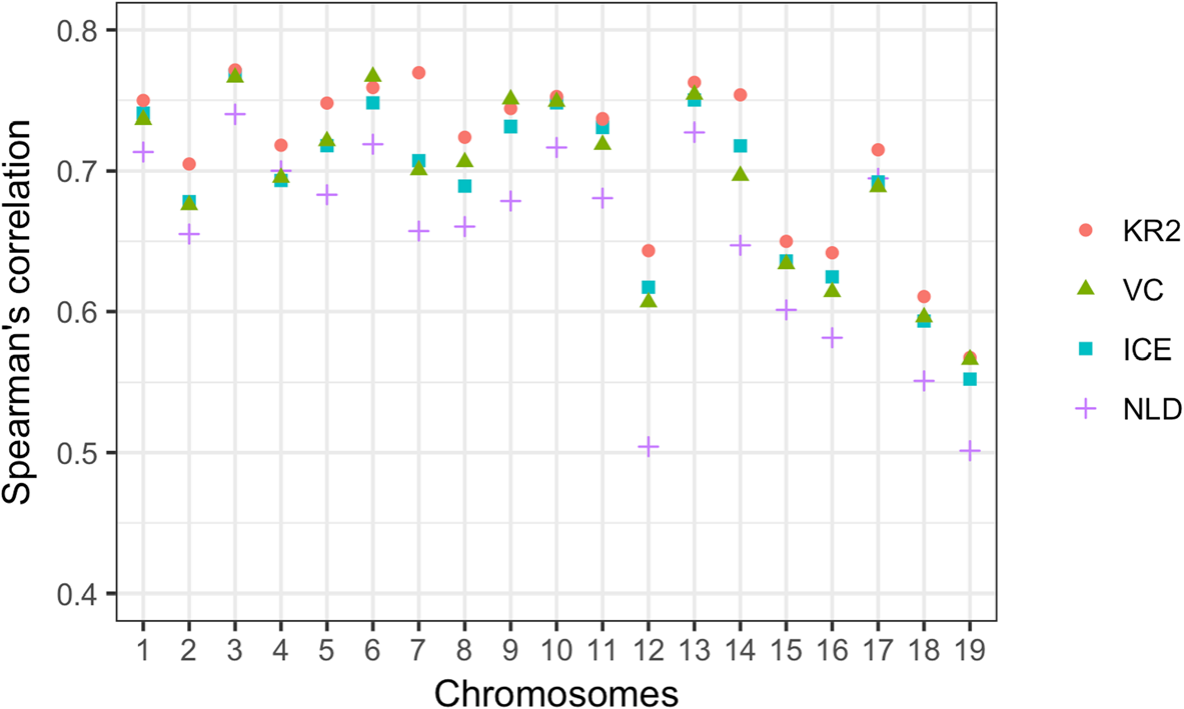
The Spearman’s correlation between normalized GAM data generated from 408 nuclear profiles at 1 Mb resolution and normalized Hi-C data. For NLD-normalized GAM data, we used ICE to normalize the corresponding Hi-C data. To compare with the ICE-normalized, VC-normalized, and KR2-normalized GAM data, we used ICE, VC, and KR to normalize the corresponding Hi-C data at 1 Mb resolution, respectively

We found KR2-normalized GAM data were more significantly correlated with the KR-normalized Hi-C data compared with the ICE or VC normalized GAM and Hi-C data (Fig. 5), which indicated that the KR-based normalization methods might be a better choice if keeping consistency between GAM and Hi-C data is of interest.

**Fig. 6:**
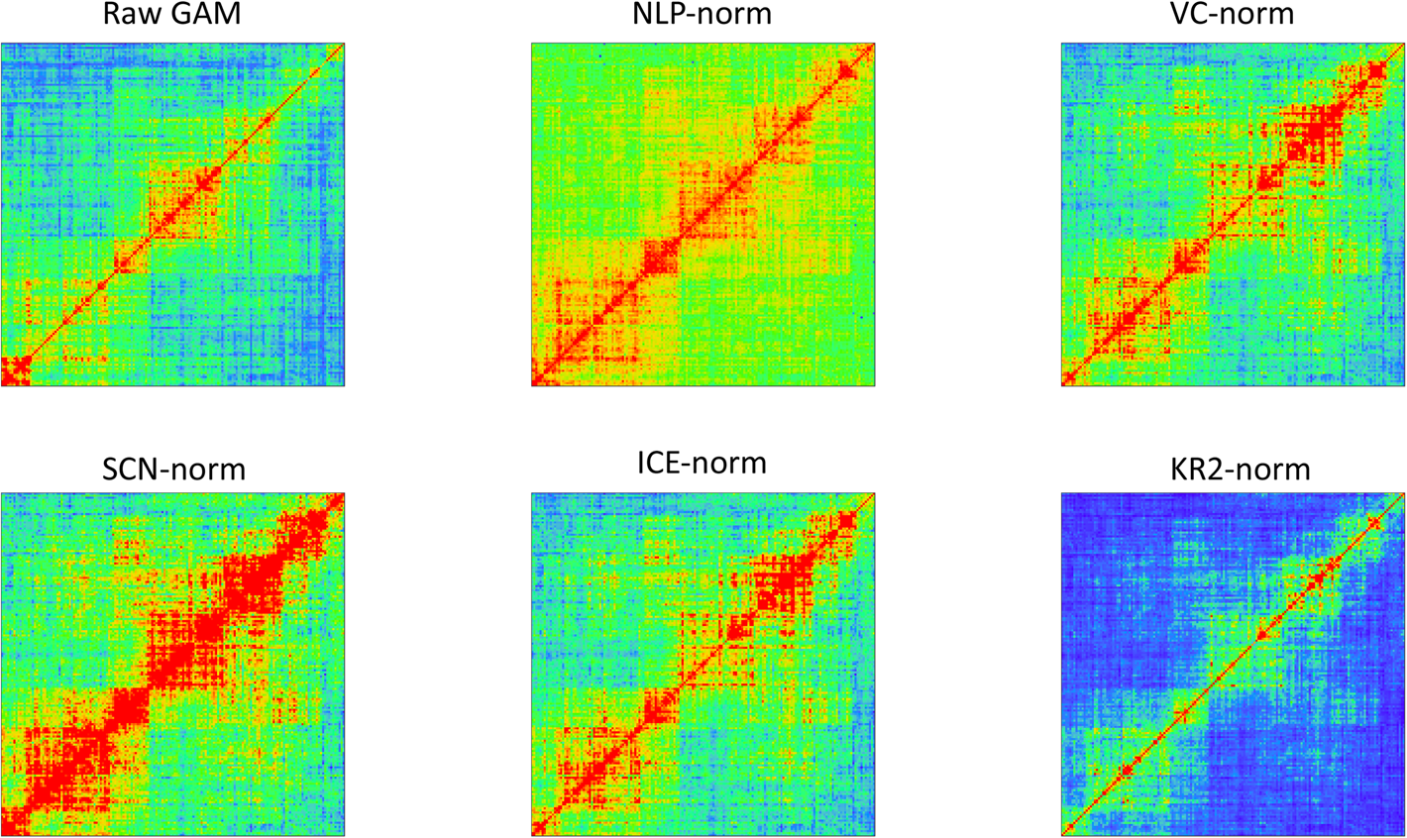
Heat maps of raw and normalized GAM contact matrices from chromosome 6 (49 Mb – 54 Mb) at 30 kb resolution

We tested whether the normalization procedures reduced or damaged the genomic interacting patterns by plotting the raw and normalized GAM contact matrices, see Fig. 6. From the six heat maps, we can observe that the five normalization methods do not compromise the interacting patterns found in the raw GAM data.

Moreover, we tested the normalization efficiency by cross-checking the normalized GAM contacts with the fluorescence in situ hybridization (FISH) data [23], that is, by comparing with the normalized FISH-detected distances between six pairs of loci: three from chromosome 2 and the other three from chromosome 11. We calculated the Spearman’s rank correlation coefficient between the FISH-detected distances and each of the average GAM contacts from raw, NLP-normalized VC-normalized, SCN-normalized, ICE-normalized, and KR2-normalized matrices; and the results are − 0.2, − 0.26, − 0.54, − 0.31, − 0.54, and − 0.49, respectively. Therefore, we can conclude that the normalization procedures not only remove systematic biases, but also make the raw GAM data more consistent with the FISH-detected distances.

## Conclusion

We found the fragment length bias, a new type of systematic bias in raw GAM data that has not been noticed before in literature. We proved that this bias existed in raw GAM data at both 1 Mb and 30 kb resolutions. We designed a new normalization method (i.e., KR2) to remove the four known biases in raw GAM data and implemented other four widely-used normalization methods in R, including NLD, VC, SCN, and ICE. We evaluated five normalization methods (i.e., NLD, VC, SCN, ICE, and KR2) at 1 Mb and 30 kb resolutions. Our evaluation results show that the five normalization methods can remove, to different extents, the four known biases; and two of them (i.e., VC and KR2) perform better than the rest. Compared with the other three methods (i.e., NLD, VC, and ICE), the KR2-normalized GAM data are more consistent with the KR-normalized Hi-C data. We also showed that the normalized GAM data (biases removed) had a higher correlation with FISH data compared with raw GAM data.

## Abbreviations

FISH: fluorescence in situ hybridization
GAM: genome architecture mapping
ICE: iterative correction and eigenvector decomposition
KR2: Knight-Ruiz 2-norm
NLD: normalized linkage disequilibrium VC vanilla coverage
NP: nuclear profile
SCN: sequential component normalization
